# LSC-like Phenotypes Aid in the Prognosis of Adult and Elderly Acute Myeloid Leukemia Patients at a Resource-Limited Health Center

**DOI:** 10.3390/cancers18091394

**Published:** 2026-04-28

**Authors:** Camila Kehl Dias, Humberto Cardoso Alves, Rafaela Bergman Rostirola, Maria Fernanda Gonçalves Meirelles Fernandes, Alexia Nedel Sant’Ana, Ana Paula Alegretti, Mariela Granero Farias, Pamela Portela da Silva, Cláudia Caceres Astigarraga, Liane Esteves Daudt, Mariana Bohns Michalowski, Alessandra Aparecida Paz, Fabrício Figueiró

**Affiliations:** 1 Programa de Pós-Graduação em Ciências Biológicas: Bioquímica, Universidade Federal do Rio Grande do Sul, Porto Alegre 90035-003, Brazil; ckdias@hcpa.edu.br (C.K.D.); alexiasantana@hcpa.edu.br (A.N.S.); 2 Departamento de Bioquímica, Instituto de Ciências Básicas da Saúde, Universidade Federal do Rio Grande do Sul, Porto Alegre 90035-003, Brazil; hcalves@hcpa.edu.br (H.C.A.); 00337669@ufrgs.br (R.B.R.); 3 Hospital de Clínicas de Porto Alegre, Porto Alegre 90035-903, Brazil; m.meirelles@edu.pucrs.br (M.F.G.M.F.); aalegretti@hcpa.edu.br (A.P.A.); mgfarias@hcpa.edu.br (M.G.F.); pporsilva@hcpa.edu.br (P.P.d.S.); castigarraga@hcpa.edu.br (C.C.A.); ldaudt@hcpa.edu.br (L.E.D.); mmichalowski@hcpa.edu.br (M.B.M.); alepaz@hcpa.edu.br (A.A.P.); 4 Programa de Pós-Graduação em Clínica Médica, Universidade Federal do Rio Grande do Sul, Porto Alegre 90035-903, Brazil; 5 Departamento de Medicina Interna, Faculdade de Medicina (FAMED), Universidade Federal do Rio Grande do Sul, Porto Alegre 90035-003, Brazil; 6 Programa de Pós-Graduação em Saúde da Criança e do Adolescente, Universidade Federal do Rio Grande do Sul, Porto Alegre 90035-903, Brazil; 7 Departamento de Pediatria, Faculdade de Medicina (FAMED), Universidade Federal do Rio Grande do Sul, Porto Alegre 90035-903, Brazil

**Keywords:** acute myeloid leukemia, tumor heterogeneity, biomarkers, prognosis, survival

## Abstract

Background/Objectives: Relapse is the greatest obstacle in treating acute myeloid leukemia (AML), occurring in 40–50% of younger patients and most elderly patients. Current immunophenotyping panels for diagnosis and detection of measurable residual disease (MRD) primarily focus on detecting blasts, thereby overlooking AML heterogeneity, which could provide valuable prognostic insights. In this context, we propose repositioning EuroFlow panels within a new set of analyses to assess leukemia stem-like phenotypes for AML prognostic monitoring. Methods: We performed a retrospective study with AML patients at the Hospital de Clínicas de Porto Alegre between 2015 and 2023. Results: Our findings highlight the relevance of leukemia stem cell-like phenotypes, particularly the CD123+, CD34+CD123+, and CD34+CD38− subpopulations, for complementary prognostication of adult and elderly patients. A higher percentage of CD123+ cells was a risk factor for both RFS (*p* = 0.04) and OS (*p* = 0.02), whereas an increase in CD34+CD123+ cells was a risk factor for RFS (*p* = 0.04) only. A higher percentage of CD34+CD38− cells was a risk factor for OS (*p* = 0.002). From diagnosis to the second monitoring, the CD34+ subpopulation was a more relevant prognostic factor in elderly individuals than in adults. CD34&CD38 most immature subpopulations may increase with poor-prognosis markers in both adults and the elderly. Conclusions: Repositioning immunophenotypic analyses may offer a cost-effective alternative for refined prognostication, particularly in healthcare centers that already have flow cytometry-based AML diagnostics.

## Simple Summary

This study aims to understand the complexity and heterogeneity of acute myeloid leukemia (AML). An in-depth analysis was conducted to assess the relevance of several biomarkers associated with a cell type believed to be the origin of leukemia and a source of treatment resistance. The presence and number of cells displaying these biomarkers were correlated with clinical characteristics, indicating whether a patient is responding to treatment. Analyzing multiple biomarkers in a single set of patients enables the selection of those that best aid in complementary prognosis prediction. Differences in the relevance of biomarkers between adult and elderly patients were highlighted. A set of analyses at diagnosis and over the first months after treatment start is proposed to improve patient management.

## 1. Introduction

Tumor heterogeneity implies that various subpopulations of cancer cells comprise the neoplastic microenvironment and interact synergistically [1]. Initially, the origin of tumor heterogeneity was explained by two models: the clonal evolution model and the cancer stem cell model [2]. These models imply that this subpopulation of cancer cells is more resistant than other populations and is responsible for treatment resistance and/or recurrence [3]. By identifying cells capable of initiating acute myeloid leukemia in immunodeficient mice, cancer stem cells were first described in leukemias, also known as leukemia stem cells (LSCs) [4]. The concept of heterogeneity has evolved to consider the relationships between different cellular populations within the tumor microenvironment (cancer cells, immune cells, and other associated cells), and the relationship between different subpopulations of each cell type [5]. When discussing intratumoral heterogeneity in the tumor cell population, factors such as surface markers, (epi)genetic abnormalities, growth, and apoptosis rates are commonly considered [6]. These characteristics contribute to cellular fitness, which is measured by the number of descendants a specific phenotype generates in a given period. The phenotypes with the highest fitness tend to resist treatment and persist, likely evolving dynamically, generating recurrence and/or relapse [7].

Acute myeloid leukemia (AML) is characterized by the uncontrolled and exaggerated growth of undifferentiated cells, called blasts, with myeloid characteristics. The remission (or non-remission) of patients with AML depends on factors such as patient age, response to therapy, and cytogenetic characteristics [8]. Hematopoietic stem cell (HSC) transplantation is also a relevant part of achieving AML remission and is considered the most potent approach to reduce relapse, especially for intermediate- and high-risk patients [9]. The detection of genetic abnormalities, mainly *FLT3-ITD*, has also been widely used and carries unfavorable prognostic significance for patients with AML [10]. However, revised protocols have reclassified *FLT3-ITD* as intermediate risk [11]. Over the years, classification and risk allocation guidelines have evolved, and a wide array of genetic abnormalities now dictate risk assessment and prognosis [12]. However, these genomic techniques are expensive and unavailable (or only partially available) in many health centers worldwide. On the other hand, multiparametric flow cytometry (MFC) is a well-established and widely used technique for diagnosis and monitoring of measurable residual disease (MRD) in acute leukemias [13]. The EuroFlow guidelines for acute leukemia diagnosis and MRD monitoring ensure reproducibility [14]. Furthermore, flow cytometry is a technique that allows sample heterogeneity appreciation, unlike most techniques used for the identification of mutations and translocations [15].

Since LSC identification, several phenotypes have been reported by flow cytometric immunophenotyping, mainly the CD34+CD38− and CD45dimCD34+CD38− phenotypes [4,16], which are associated with greater undifferentiation. Other previously reported and relatively accepted markers are the lack of CD117, also known as c-KIT, a proto-oncogene, and positivity for CD123, CD96, CLL-1, and/or TIM-3 [17]. The expression of CD123 in AML LSCs is also associated with the *FLT3-ITD* mutation, including the CD34+CD38−CD123+ phenotype [17], which, in turn, correlates with increased recurrence and decreased overall survival [18]. Other characteristics, such as HLA-DR-, CD33+, and CD25−/+, have been proposed for LSCs but are not yet fully established [19]. Lastly, some markers, such as CD36, are not necessarily associated with LSCs but correlate with infiltration in other sites and relapse [20]. Recent clinical approaches to account for the presence/abundance of LSC have shown the prognostic value of the LSC burden, especially in AML. ELN22 guidelines suggest monitoring of LSCs is relevant, but specific phenotypes are not cited; nor is burden or patterns between diagnosis and early treatment [11]. Relevant trials in Europe (HOVON-SAKK) have been conducted proposing an LSC tube and validating the use of LSC burden as a prognostic factor associated with survival [21]. However, this strategy requires the establishment of a new MFC tube, and there is no similar available data for Latin American patients, especially patients who are engaged in different stratification and treatment protocols, mostly due to financial constraints.

Given that AML heterogeneity hampers patient response to treatment, more and more strategies that account for this heterogeneity must be developed [22]. Hence, in this work, we propose repurposing EuroFlow antibody panels to complement prognostication at diagnosis and monitoring in AML. This new, practical, and easily implementable approach aims to identify subpopulations of blasts or immature cells and to associate their presence/abundance with prognosis and survival. This study introduces new cost-effective strategies for centers that already use flow cytometry for leukemia diagnosis and follow-up. This is the first study to propose LSC-like phenotypes detection in Latin American patients in a resource-limited setting.

## 2. Materials and Methods

In this work, we conducted a retrospective study of patients diagnosed with acute myeloid leukemia (with the exception of acute promyelocytic leukemia) over the course of seven years at the Hospital de Clínicas de Porto Alegre (HCPA), a public hospital in southern Brazil.

### 2.1. Patient Consent

This study followed the principles of the Declaration of Helsinki. Authorized consent was acquired from patients who were still in follow-up when the data for the study were collected. Consent was given through a telephone call, and an informed consent form was sent to the participants via e-mail. The General Law of Data Protection (LGPD) from Brazil was taken into consideration when the data were collected, and all the information was anonymized to comply with the LGPD. The study was approved by the Hospital de Clínicas de Porto Alegre ethics committee and Plataforma Brasil, a national ethics committee (codes 2021-0512 and 53043421.7.0000.5327, respectively).

### 2.2. Patients

All adult and elderly patients diagnosed with AML at HCPA from February 2015 to January 2023 with available bone marrow immunophenotypic data were included in this study, with 99 patients in total (57 adults and 42 elderly individuals). Adult (≥18 and <60) and elderly (≥60) patients were analyzed separately. In Table S1, detailed information regarding the sample size of each analysis and the clinical characteristics of the patients can be found. The 7 + 3 protocol was applied to adults, and azacitidine or cytarabine was applied to elderly patients (venetoclax not available). Most up-to-date stratification guidelines and treatment protocols for AML could not have been applied since all patients were treated according to what is offered by the Brazilian public health system. The first MRD monitoring was on day 30 (from the start of treatment), whereas the second was on day 90 (approximately 90). These dates were standardized across the cohort (30 ± 2 days and 90 ± 7 days/mean ± SEM).

### 2.3. Risk Stratification

Patients were stratified into risk groups according to an adapted version of the European LeukemiaNet (ELN) 2017 criteria [23], adjusted for the availability of molecular markers at our institution during the period described. The following criteria were applied:

• Favorable Risk: Defined by the presence of core-binding factor (*CBF*) rearrangements, specifically t(8; 21)(q22; q22.1) or inv(16)(p13.1q22)/t(16; 16)(p13.1; q22), identified via conventional karyotype.
• Intermediate Risk: Comprised patients with a normal karyotype (NK) or cytogenetic abnormalities not categorized as favorable or adverse. In the absence of *NPM1* mutational status (implemented locally in August 2024), all NK patients without *FLT3-ITD* mutations were assigned to this group.
• Adverse Risk: Defined by high-risk cytogenetics (e.g., complex or monosomal karyotypes, −5, −7, del(5q), and 11q23.3 rearrangements) and the presence of *FLT3-ITD* detected by PCR.

Because the FLT3-ITD allelic ratio was not quantitatively determined, any qualitative detection of the *FLT3-ITD* mutation was considered an adverse finding for the purposes of this classification.

### 2.4. Immunophenotyping Files Analysis

Flow cytometry “.fcs” files from the Hematology and Flow Cytometry Unit of the HCPA laboratory were reanalyzed (all files were reviewed by the same operator), and the findings were in agreement with the routine diagnostic reports from the Flow Cytometry unit. Only bone marrow immunophenotyping records were selected for this study. The antibody panels applied at diagnosis and MRD monitoring are listed in Table S2. They were performed as recommended by the Euroflow consortium from the European Scientific Foundation for Laboratory Hemato Oncology [14,24]. For the analysis of the diagnosis files, tube 3 (Table S2) was used to evaluate the CD34+, CD36+, and CD34+CD36+ subpopulations of blasts/immature cells; tube 5 (Table S2) was used for the CD34&CD38 analysis; and tube 6 (Table S2) was used for the CD123+ and CD34+CD123+ analyses. For MRD monitoring file analysis, tube 1 from the MRD panel (Table S2) was used to evaluate the CD34+ and CD34&CD38 subpopulations of blasts/immature cells. The panel is performed hierarchically; therefore, once a diagnosis is established, subsequent panels are not performed, and not all patients have data from all tubes. To normalize MFI values and reduce interpatient heterogeneity, we used the MFI ratio between the subpopulation positive for each marker and the respective negative subpopulation (e.g., the CD34+ CD117 MFI ratio = CD117 MFI of CD34+ blasts/CD117 MFI of CD34− blasts), computed using the median MFI. Further information on all the parameters evaluated for each analysis and how the MFI ratio was calculated can be found in Table S3. Infinicyt software (RRID:SCR_026033) version 1.7 (BD Biosciences, San Jose, CA, USA) was used for all analyses.

### 2.5. Gating Strategy

First, debris and doublets were excluded. Secondly, eosinophils, neutrophils, monocytes, lymphocytes, and erythroblasts were separated from the immature cells/blasts, using FSC and SSC parameters and CD45, CD117, and HLA-DR expression. When tube six was used from the diagnostic panel (Table S2), it was also possible to identify and separate basophils and mast cells. For our subpopulation analyses, we selected medium- to low-SSC and CD45dim cells that remained after all the cell populations described above were separated and were assigned blast/immature cells. The expression of CD117 and HLA-DR by these cells was also considered, especially in relation to clinical reports issued for each patient at each time point (diagnosis or monitoring). We then identified blast/immature cell subpopulations: CD34+, CD36+, CD34+CD36+, CD123+, CD34+CD123+, and CD34+CD38−, CD34+CD38+, CD34−CD38+, and CD34−CD38−. Lymphocytes were used as the negative control for the positivity setting of each marker in blasts/immature cells. The median fluorescence intensity (MFI) was calculated as the ratio between the MFI of the marker-positive population and that of the corresponding negative population. For single markers, this was defined as CD34+ MFI divided by CD34− MFI and similarly applied to CD36+ and CD123+ subpopulations. For combinations of markers, the MFI of CD34+CD36+ blasts was divided by the MFI of other SSClowCD45dim blasts, and the MFI of CD34+CD123+ cells was calculated using the same approach. For CD34&CD38 subpopulations, MFI was determined as CD34+CD38+ MFI, for example, divided by CD34−CD38− MFI. Further details on the analysis are provided in Figure S1 and Table S3.

Only CD34+ and CD34&CD38 analyses were performed at the time of diagnosis, and at the time of the first and second rounds of monitoring (when available). CD36 and CD123 analyses were performed only at the time of diagnosis.

### 2.6. Data Analysis

All statistical analyses were performed via Prism software version 10 (RRID:SCR_002798) (GraphPad software, Boston, MA, USA). The Shapiro–Wilk test and QQ plots were used to verify the assumption of normality of all the variables assessed. For normally distributed data, a *t*-test (if standard deviations were too different, Welch’s correction was applied) or one-way ANOVA was used; for nonnormally distributed data, Mann–Whitney or Kruskal–Wallis tests were used. Specifically, for CD34 and CD38 analysis at diagnosis, multiple t tests with the Holm–Šídák method were used. ROC curves were generated with the Wilson/Brown method and a 95% confidence interval. For diagnosis/day-30/day-90 temporal analyses, simple linear regressions were calculated, and the difference between slopes was tested. Spearman’s test was used for correlation analyses. Finally, Cox regressions were calculated for all parameters analyzed at the time of diagnosis, normalized by age and biological sex; the method for tie estimation used was exact for small numbers of ties or Efron’s approximation otherwise.

## 3. Results

### 3.1. Different LSC-like Phenotypes Are Associated with an Increased Risk of Relapse or Death

First, we analyzed whether the studied subpopulations had any associations with relapse-free survival (RFS) or overall survival (OS). All analyses were normalized by sex and age and were performed at five-year, three-year, and one-year intervals (with different sample sizes) for all parameters at diagnosis (blast subpopulation percentages and MFI ratios, see Table S3 for details).

When evaluating five-year OS, the percentage of CD123+ blasts (from total blasts) and the percentage of CD34+CD38− blasts (from both total cells and total blasts) presented hazard ratios greater than 1. Thus, higher percentages of these subpopulations at diagnosis represent an increased risk of death for patients. For five-year RFS, we observed that higher CD123+ and CD34+CD123+ blast percentages (from total blasts) were associated with shorter RFS (Table 1). Cox regression analysis was not possible for the five-year RFS in the CD36+ or CD36+CD34+ subpopulations due to the small number of patients with relapse data in these subgroups. All other parameters not mentioned here (Table 1) were nonsignificant for five-, three-, or one-year OS and RFS.

Furthermore, we performed correlation analyses for all factors (percentages and MFI ratios) against age, RFS, and OS for five-, three-, and one-year follow-ups (all significant results can be found in Table S4). Some five-year correlation analyses presented significant *p*-values and moderately strong to strong correlations, especially Age with CD123+% from total blasts, with the CD123 MFI ratio from CD123+ blasts, and with CD34+CD123+% from total blasts (Spearman’s r: −0.692, 0.738, and −0.800, respectively). Additionally, the RFS and the NG2 MFI ratio from CD34+CD38− blasts were significantly correlated over the five-year period (Spearman’s r: 0.852). During the three-year period, only age and the CD123 MFI ratio among CD34+CD123+ cells showed a moderately strong correlation (Spearman’s r: 0.612).

For the three-year period, a greater CD34+CD38− blast percentage (from total cells) was associated with shorter OS, which remained significant. On the other hand, a higher CD117 MFI ratio was associated with greater OS, specifically in the CD34+ subpopulation. Both the CD117 MFI ratio in CD34+ blasts and the percentage of CD34+CD38− cells among total cells were significant in the Cox regression analyses, with age as another significant factor (elderly patients had an increased risk of death). Higher CD123+ blast percentage remained associated with RFS, in the three-year period, with male patients having an increased risk of relapse.

Finally, we performed Cox regression analyses for a one-year follow-up since the median survival and time until relapse were mainly under 12 months for adults and elderly individuals (Figure S2a–f). Regarding OS, the CD117 MFI ratio in CD34+ blasts remained a significant protective factor, and age also had a significant effect, as observed in the three-year analysis. The percentage of CD34+CD38− blasts among total cells also remained a significant predictor of OS. Higher percentages of this subpopulation were associated with increased risk of death, as shown in three- and five-year analyses, with age also being a significant factor. Furthermore, HLA-DR MFI ratio in the CD34+CD123+ subpopulation was associated with RFS in the one-year follow-up. The higher the MFI ratio in this subpopulation, the greater the RFS in AML patients. On the other hand, many other factors were observed to have a negative effect on RFS: the CD34 MFI ratio from CD34+CD123+ and CD34−CD38+ blasts, and the NG2 MFI ratio from CD34+CD38+ blasts (Table 1).

### 3.2. Different Subpopulation Frequencies and Marker Expression at Diagnosis Are Associated with Risk, Treatment Response, and Relapse

Considering that some phenotypes studied were associated with lower OS and RFS, with some markers showing protective effects, we sought to investigate whether they would also correlate with prognostic factors such as the presence of the FLT3-ITD alteration, the risk assigned at diagnosis, the achievement of complete morphological remission (CR) by day 30 of treatment (<5% blasts in the bone marrow), and relapse at any time during follow-up (the sample size of elderly patients who relapsed was too small for statistical analysis).

#### 3.2.1. CD36+ Subpopulations

**Figure 1:**
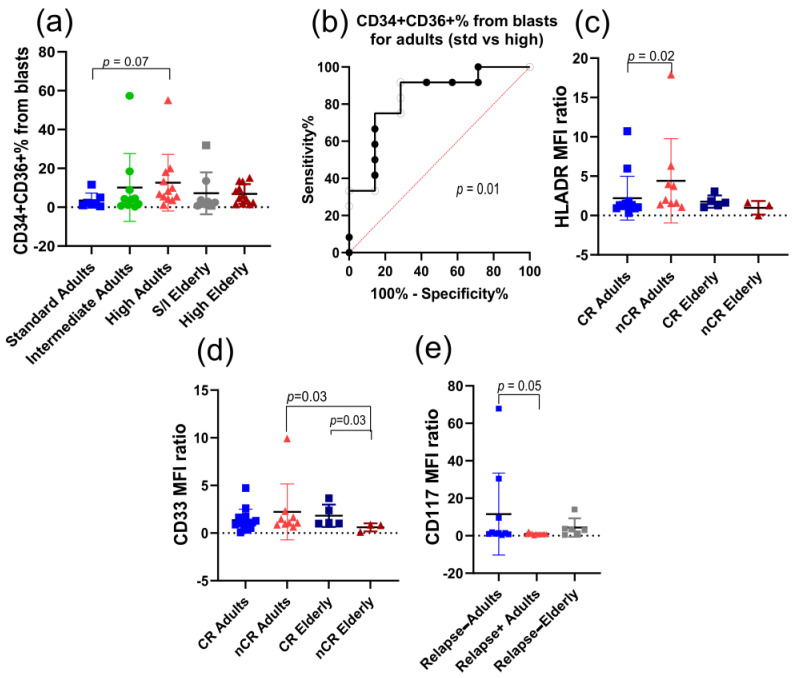
CD34+CD36+ subpopulation associations with prognosis parameters at diagnosis. (**a**) CD34+CD36+ blast percentage from total blasts (Kruskal–Wallis test between adult risks *p* = 0.05, Dunn’s post-test between adults from standard- and high-risk *p* = 0.07); (**b**) ROC curve for CD34+CD36+ blast percentage comparing standard- and high-risk adults (*p* = 0.01); (**c**) HLA-DR MFI ratio from CD34+CD36+ blasts (Mann–Whitney test between CR and nCR adults *p* = 0.02); (**d**) CD33 MFI ratio from CD34+CD36+ blasts (Mann–Whitney test between CR and nCR elders *p* = 0.03); (**e**) CD117 MFI ratio from CD34+CD36+ blasts (Mann–Whitney test between relapse and non-relapse adults *p* = 0.05). CR: complete remission; nCR: incomplete remission.

We observed a gradual increase in CD34+CD36+ cells in relation to total blasts according to adult patient risk, although this increase was not significant (Figure 1a). Furthermore, the ROC curve comparing these two groups revealed that it is possible to differentiate between standard- and high-risk patients according to the CD34+CD36+ blast percentage (Figure 1b). In addition, adult patients who did not achieve complete remission presented a higher HLA-DR MFI ratio than did adults who achieved complete remission by day 30 (Figure 1c). Additionally, elderly patients who did not achieve complete remission had lower CD33 MFI ratios than did elderly patients who achieved remission (Figure 1d). Finally, adult patients who did not relapse presented higher CD117 MFI ratios than adult patients who relapsed, although the difference was not significant (Figure 1e).

When we examined the CD36+ subpopulation, we observed that intermediate-risk adults had a higher CD34 MFI ratio than high-risk adults (Figure S3a). Compared with standard-risk patients, intermediate-risk adult patients also had higher CD117 MFI ratios (Figure S3b). Adult patients who relapsed had lower CD36 MFI ratios and higher CD34 MFI ratios, in comparison to adults who did not relapse (Figure S3c,d).

#### 3.2.2. CD123+ Subpopulations

**Figure 2:**
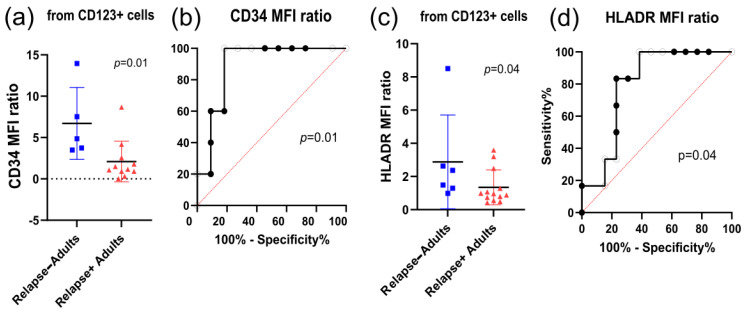
CD123+ subpopulation association with prognosis parameters at diagnosis. (**a**) CD34 MFI ratio from CD123+ blasts (Mann–Whitney test between relapse and non-relapse adults, *p* = 0.01); (**b**) ROC curve for the CD34 MFI ratio from CD123+ blasts comparing relapse and non-relapse adults (*p* = 0.01); (**c**) HLA–DR MFI ratio from CD123+ blasts (Mann–Whitney test between relapse and non-relapse, *p* = 0.04); (**d**) ROC curve for the HLA–DR MFI ratio from CD123+ blasts comparing relapse and non-relapse adults (*p* = 0.04).

CD123 is a well-studied marker of LSCs; therefore, the CD123+ and CD34+CD123+ subpopulations were also analyzed at diagnosis. Adult patients who did not relapse had higher CD34 MFI ratios in CD123+ cells than those who did relapse (Figure 2a). This marker allowed excellent differentiation between these groups (Figure 2b). In addition, adult patients who did not relapse had higher HLA-DR MFI ratios than did adult patients who relapsed (Figure 2c). This characteristic provided satisfactory differentiation between the relapsed and non-relapsed groups (Figure 2d). Further, the CD34 MFI ratios decreased gradually with risk in adult patients (Figure S3e), with excellent differentiation between standard- and high-risk groups (Figure S3f). Finally, for the CD34+CD123+ subpopulation, no significant changes in percentage or marker expression were observed.

#### 3.2.3. CD34&CD38 Subpopulations

**Figure 3:**
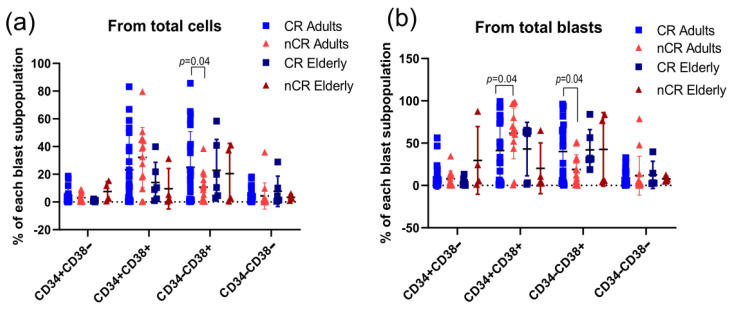
CD34&CD38 subpopulations associations with prognosis parameters at diagnosis. (**a**) CD34&CD38 subpopulations percentage from total cells (multiple *t*-tests by the Holm–Šídák method, *p* = 0.04 for CD34−CD38+ between CR and nCR adults); (**b**) CD34&CD38 subpopulations percentage in relation to total blasts (multiple *t*-tests by h the Holm–Šídák method, *p* = 0.04 for CD34+CD38+ and CD34−CD38+ between CR and nCR adults). CR: complete remission; nCR: incomplete remission.

Given the extensive discussion in the literature on whether AML LSCs are exclusively CD34+CD38−, we also investigated all four subpopulations: CD34+CD38−, CD34+CD38+, CD34−CD38+, and CD34−CD38−. We observed that adult patients who achieved complete remission by day 30 of treatment had a higher percentage of CD34−CD38+ blasts relative to total cells and total blasts (Figure 3a,b). Additionally, concerning total blasts, adult patients who did not achieve remission had a higher percentage of CD34+CD38+ blasts (Figure 3b). No similar patterns were observed for elderly patients.

### 3.3. Temporal Analysis of Subpopulations from Diagnosis to the First and Second Monitoring Reveals Differential Patterns Between Patients with Good and Poor Prognoses

After observing that some subpopulations were more abundant in patients with worse prognosis at diagnosis or presented different immunophenotypes, we wondered whether these subpopulations would still show differential patterns throughout post-diagnosis monitoring. On average, the first monitoring after treatment started occurred on day 30, and the second occurred on day 90. Only temporal analyses of the CD34+ and CD34+CD38−, CD34+CD38+, CD34−CD38+, and CD34−CD38− subpopulations were possible because of marker availability on the Euroflow MRD panel for AML.

**Figure 4:**
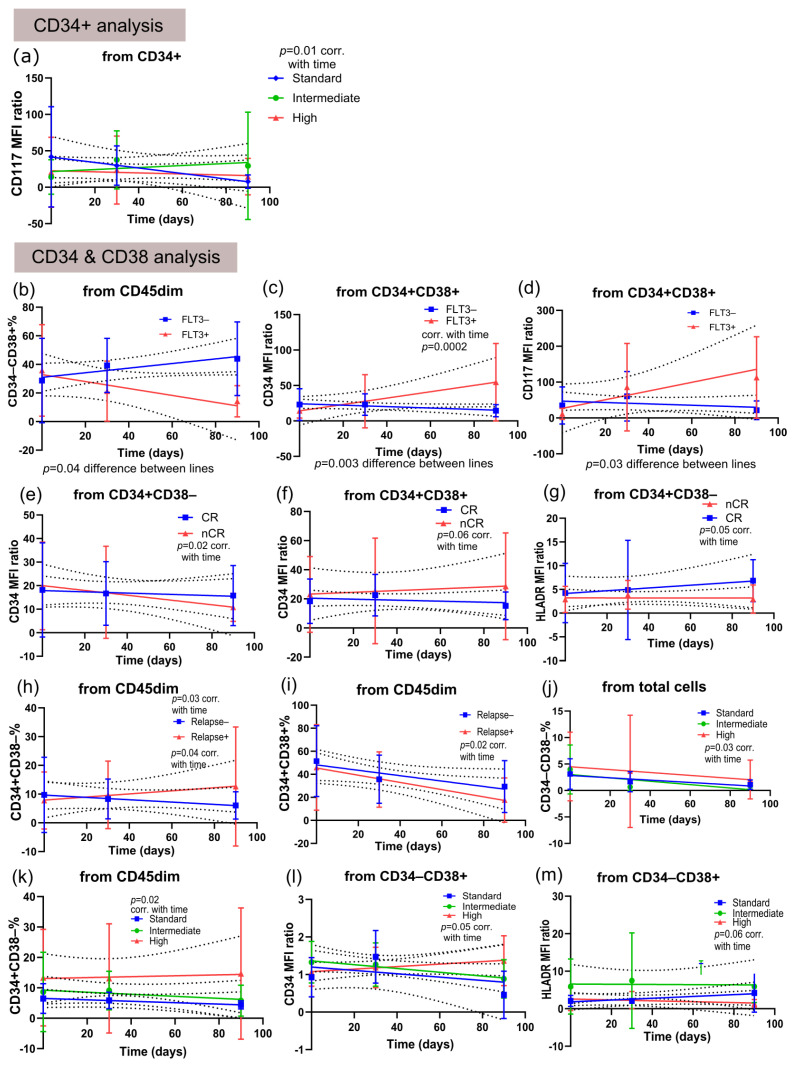
Temporal analyses from diagnosis to second monitoring of subpopulations of CD45dim cells from adult patients. (**a**) CD117 MFI ratio from CD45dimCD34+ cells (for standard-risk correlation with time *p* = 0.01); (**b**) CD34–CD38+ percentage in relation to CD45dim cells (simple linear regression showed that FLT3− and FLT3+ lines are different *p* = 0.04); (**c**) CD34 MFI ratio from CD45dimCD34+CD38+ cells (simple linear regression showed that FLT3− and FLT3+ lines are different *p* = 0.003/for FLT3+ correlation with time *p* = 0.0002); (**d**) CD117 MFI ratio from CD45dimCD34+CD38+ cells (simple linear regression showed that FLT3− and FLT3+ lines are different *p* = 0.03); (**e**) CD34 MFI ratio from CD45dimCD34+CD38− cells (for nCR correlation with time *p* = 0.02); (**f**) CD34 MFI ratio from CD45dimCD34+CD38+ (for nCR correlation with time *p* = 0.06); (**g**) HLA-DR MFI ratio from CD45dimCD34+CD38− cells (for nCR correlation with time *p* = 0.05); (**h**) CD34+CD38− percentage from CD45dim cells (for relapse-correlation with time *p* = 0.03 and for relapse+ *p* = 0.04); (**i**) CD34+CD38+ percentage from CD45dim cells (for relapse+ correlation with time *p* = 0.02); (**j**) CD34−CD38− percentage from CD45dim cells (for high-risk correlation with time *p* = 0.03); (**k**) CD34+CD38− percentage from CD45dim cells (for standard-risk correlation with time *p* = 0.02); (**l**) CD34 MFI ratio from CD45dimCD34−CD38+ cells (for high-risk correlation with time *p* = 0.05); (**m**) HLA-DR MFI ratio from CD45dimCD34−CD38+ (for high-risk correlation with time *p* = 0.06).

In the CD34+ subpopulation analysis of adult patients, only standard-risk patients presented a decrease in the CD117 MFI ratio that correlated with time (Figure 4a). In the analyses of CD34&CD38 subpopulations, various associations were observed. Compared to *FLT3*-ITD-positive patients, FLT3-*ITD*-negative adult patients presented an increase in the CD34−CD38+ subpopulation (Figure 4b). In contrast, the *FLT3-ITD*-positive patients presented increases in the CD34 and CD117 MFI ratios in the CD34+CD38+ subpopulation over time (Figure 4c,d). Adult patients who did not achieve complete remission presented a decrease in the CD34 MFI ratio among CD34+CD38− cells but presented a non-significant increase in the CD34 MFI ratio among CD34+CD38+ cells (Figure 4e,f). In contrast, adult patients who achieved complete remission after 30 days of treatment presented a non-significant increase in the HLA-DR MFI ratio among CD34+CD38− cells (Figure 4g). Notably, the percentage of CD34+CD38− cells within the CD45dim population was strongly associated with relapse: adult patients who relapsed showed an increase in this subpopulation, whereas those who did not relapse showed a decrease (Figure 4h). The CD34+CD38+ subpopulation also presented a significant decrease (from CD45dim) in adult patients who relapsed, although non-relapsed patients also presented a nonsignificant decreasing pattern (Figure 4i). High-risk adult patients were the only group that presented a significant decrease in the percentage of CD45dimCD34−CD38− cells among total cells (Figure 4j). Furthermore, standard-risk adult patients were the only group that presented a significant decrease in CD34+CD38− cells from CD45dim cells (Figure 4k). Additionally, high-risk adult patients presented an increase in the CD34 MFI ratio and a reduction in the HLA-DR MFI ratio from CD34−CD38+ cells, although both were non-significant (Figure 4l,m).

**Figure 5:**
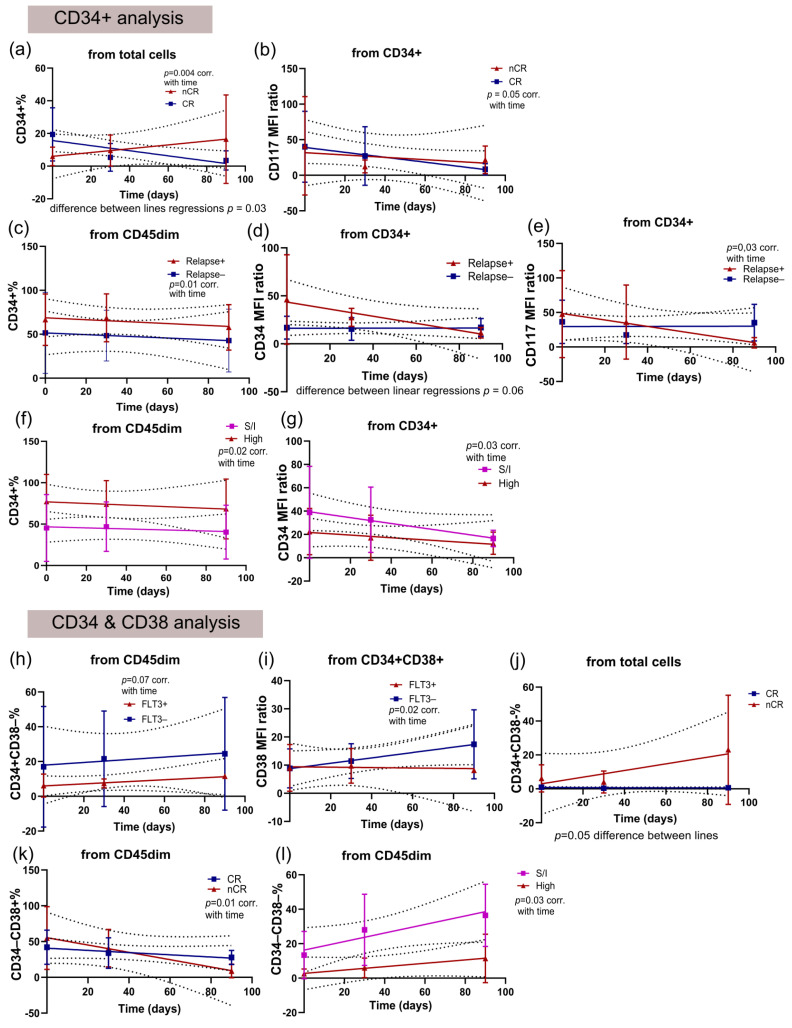
Temporal analyses from diagnosis to second monitoring of subpopulations of CD45dim cells from elderly patients. (**a**) CD34+ percentage in relation to total cells (simple linear regression showed that nCR and CR lines are different *p* = 0.03/for nCR correlation with time *p* = 0.004). (**b**) CD117 MFI ratio from CD45dimCD34+ cells (for CR correlation with time *p* = 0.05). (**c**) CD34+ percentage from CD45dim cells (for relapse- correlation with time *p* = 0.01). (**d**) CD34 MFI ratio from CD45dimCD34+ cells (simple linear regression showed that relapse− and relapse+ *p* = 0.06). (**e**) CD117 MFI ratio from CD45dimCD34+ (for relapse+ correlation with time *p* = 0.03). (**f**) CD34+ percentage from CD45dim cells (for high-risk correlation with time *p* = 0.02). (**g**) CD34 MFI ratio from CD45dimCD34+ cells (for S/I-risks correlation with time *p* = 0.03). (**h**) CD34+CD38− percentage from CD45dim cells (for FLT3+ correlation with time *p* = 0.07). (**i**) CD38 MFI ratio from CD45dimCD34+CD38+ (for FLT3− correlation with time *p* = 0.02). (**j**) CD34+CD38− percentage from total cells (simple linear regression between nCR and CR *p* = 0.05). (**k**) CD34−CD38+ percentage from CD45dim cells (for nCR correlation with time *p* = 0.01). (**l**) CD34-CD38- percentage from CD45dim cells (for high-risk correlation with time *p* = 0.03).

The temporal analyses revealed strikingly different patterns for elderly patients than for adult patients. Essentially, the CD34+ subpopulation presented several significant patterns. For example, patients who did not achieve complete remission presented an increase in CD45dimCD34+ cells (from total cells), whereas patients who achieved remission presented a decrease in these cells (Figure 5a). Elderly patients who achieved remission presented a non-significant decrease in the CD117 MFI ratio among CD34+ cells (Figure 5b). Furthermore, elderly patients who did not relapse presented a decrease in the percentage of CD34+ cells among CD45dim cells (Figure 5c). Patients who relapsed presented a reduction in the CD34 (non-significant) and CD117 (significant) MFI ratios among CD45dimCD34+ cells (Figure 5d,e). In contrast, high-risk elderly patients showed a decrease in CD34+ cells from the CD45dim subpopulation (although the percentages remained consistently higher), whereas standard- and intermediate-risk patients showed a decrease in the CD34 MFI ratio from this subpopulation (Figure 5f,g).

While the CD34+ subpopulation appears to be of greater importance in elderly patients, some observations on the CD34&CD38 subpopulations were also noteworthy. *FLT3-ITD*-positive elderly patients showed a non-significant increase in the percentage of CD34+CD38− cells among CD45dim cells, whereas FLT3-ITD-negative patients showed consistently high percentages of this subpopulation over time (Figure 5h). While *FLT3-ITD*-negative patients presented increased CD38 MFI ratios among CD34+CD38+ cells (Figure 5i). Elderly patients who did not achieve complete remission demonstrated a non-significant increase in CD45dimCD34+CD38− cells from total cells compared to patients who achieved complete remission, with a much higher area under the curve (Figure 5j). Furthermore, patients who did not achieve complete remission also presented a decrease in CD34−CD38+ cells from CD45dim cells (Figure 5k). Similarly, high-risk patients demonstrated an increase in CD34−CD38− cells over time (from CD45dim), even with a much smaller area under the curve than standard- and intermediate-risk patients did (Figure 5l).

### 3.4. Patients Who Underwent Allo-HCT Presented Differences in Subpopulation Frequency and Phenotypes at Diagnosis

**Figure 6:**
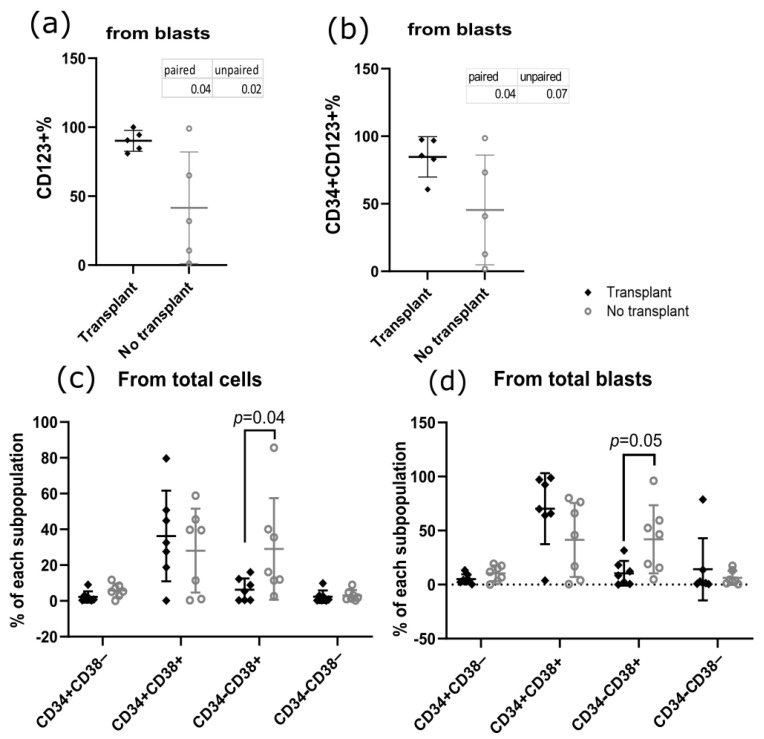
CD123+, CD34+123+, and CD34&CD38 subpopulations comparison at diagnosis in a transplant-patient subset. (**a**) CD123+ percentage from blasts (paired *t* test *p* = 0.04/unpaired *t* test *p* = 0.02); (**b**) CD34+123+ percentage from blasts (paired *t* test *p* = 0.04/unpaired *t* test *p* = 0.07); (**c**) CD34&CD38 subpopulations percentage in relation to total cells (two-way ANOVA followed by Sidak’s multiple comparisons test, *p* = 0.04 for CD34−CD38+); (**d**) CD34&CD38 subpopulations percentage in relation to total blasts (two-way ANOVA followed by Sidak’s multiple comparisons test, *p* = 0.05 for CD34−CD38+).

After noting that potential LSC phenotypes correlate with several prognostic factors and their persistence, we sought to investigate whether any of the studied phenotypes could also predict the indication for transplantation. First, we observed that transplanted patients presented increased three-year OS and a greater blast percentage at the first monitoring, while there were no differences in RFS or the percentage of blasts at the end of induction (Figure S2g–j). Owing to the small sample size of transplanted patients compared with the whole study sample size (Table S1), we present a paired analysis by sex and age with patients who did not undergo transplant and survived. The percentage of CD123+ blasts at diagnosis was increased in patients who later underwent transplantation (Figure 6a), as was the percentage of CD34+CD123+ blasts (Figure 6b), in comparison to patients of similar age and sex who survived and did not require transplant. Finally, for the CD34&CD38 subpopulations, we observed that patients who later underwent transplantation presented lower percentages of CD34−CD38+ blasts at the time of diagnosis, for total cells (significant) and total blasts (non-significant) (Figure 6c,d).

## 4. Discussion

In investigating several potential LSC phenotypes in acute myeloid leukemia patients, we unveiled interesting patterns in the MFC diagnosis data. First, more than one phenotype was negatively associated with OS and RFS in the various periods analyzed, namely, CD34+CD38−, CD123+, and CD34+CD123+.

The percentage of CD34+CD38− blasts has been previously associated with shorter OS, which is in line with our findings [21,25]. Owing to the greater presence of LSCs in the CD34+CD38− more immature compartment, the first studies in the field considered that LSCs arise from normal stem cells. Research has demonstrated that LSCs do not necessarily originate from HSCs and can be present in CD34-negative compartments [26]. LSCs are most commonly found in the most immature population among those present in the blasts of each patient, but not exclusively, since the same patient can present more than one population of LSCs [27]. Especially in leukemias, the heterogeneity of populations is exacerbated by the presence of different clones at different maturation stages. Thus, we observed a shift between the percentages of CD34+CD38+ and CD34−CD38+ cells at the time of diagnosis in adult patients who did and did not achieve CR, and this same pattern was observed in patients who later underwent transplantation, indicating that the other compartments can also yield relevant information. Furthermore, it is still unclear how LSCs persist during treatment and what happens to the initial phenotypes presented at diagnosis until recurrence [5]. The expression of CD34 and NG2 in all CD34&CD38 populations, except the double-negative population, was associated with shorter RFS. NG2 expression being associated with shorter RFS was expected due to prior observations of the relevance of NG2 in AML [28]; nonetheless, it is interesting that this association was observed only in the CD34+CD38+ subpopulation.

Furthermore, the CD123+ phenotype presented equal, if not greater, robustness in dictating prognosis than the CD34+CD123+ phenotype did, which is somewhat contrary to current literature trends focusing on the CD34+CD38−CD123+ phenotype [29]. The CD123+ percentage was the only parameter analyzed in this study to be negatively associated with both OS and RFS, an exceptional finding, as it is rare for a single marker to offer such valuable prognostic insight, and aligns with the findings of another study [30]. Additionally, patients who underwent transplantation presented higher percentages of both CD123+ and CD34+CD123+ blasts at diagnosis. Interestingly, CD123 has been proposed as a good marker for MRD [31], underscoring its multiple applications in AML follow-up; however, we could not analyze its expression at diagnosis in this cohort.

In our work, CD36 appeared to be a good marker for diagnosis only when combined with CD34, as a higher percentage of CD34+CD36+ blasts was observed in high-risk patients than in standard-risk patients. This is somewhat at odds with recent observations on the value of CD36 [20]; nonetheless, differences in risk stratification and treatment must be considered. However, other markers analyzed stood out more.

Other interesting patterns emerged when the CD34+ and CD34&CD38 subpopulations were evaluated from the time of diagnosis through the first and second monitoring rounds. The CD34+ subpopulation presented more pronounced alterations in elderly individuals than in adults. Elderly patients who did not achieve complete remission presented an increase in the percentage of CD34+ cells. This subpopulation remained consistently elevated from diagnosis to the second monitoring in high-risk elderly patients, and those who relapsed. Furthermore, the percentage of CD34+CD38− cells increased in elderly patients who did not achieve complete remission. It has recently been reported that detection of CD34+CD38− (with other aberrancies) at diagnosis and, especially, after three cycles of treatment with hypomethylating agents correlates with lower OS and a higher risk of relapse [32].

On the other hand, adult *FLT3-ITD*-positive patients presented a decrease in the percentage of CD34−CD38+ cells and an increase in CD34+CD38+ CD34 and CD117 MFI ratios. It has been reported that CD34+CD38−CD123+ LSCs are primarily *FLT3-ITD*-positive, highlighting the association between genotype and phenotype [33]. Finally, in adults, the CD34+CD38− subpopulation increased in patients who relapsed and decreased in patients who did not relapse and/or were classified as standard risk. The CD34+CD38− phenotype is widely recognized to be associated with LSCs and poor prognosis [34]. It has also been established that this phenotype, especially with CD123 positivity, can be used in MRD detection [35]. However, little is discussed on how the follow-up of these subsets can provide useful insights for clinicians, especially until the second monitoring, as described here. We highlight how MFC analysis yields different results for adults and elderly patients; it has also been proposed as the key technique for MRD detection, especially in elderly patients [36].

Regarding the expression of CD117 and HLADR in the different phenotypes analyzed, an increase in HLA-DR MFI ratios correlates in general with better prognosis, while the results with CD117 MFI ratios are mixed. The decrease in CD117 MFI ratio in CD34+ cells throughout time was observed in elderly patients who relapse, while the same pattern was observed for adult standard-risk patients; CD117 expression has previously been linked to poor prognosis [37]. The correlation between CD117 and HLA-DR expression in AML has also been extensively studied [38]. Indeed, HLA-DR positivity, including CD117, CD34, and CD123 positivity, has been linked to poor prognosis in elderly AML patients [39]. However, in our study, adult patients who relapsed had a lower HLA-DR MFI ratio in CD123+ cells at diagnosis, while it also had a protective effect on one-year OS and RFS in CD34+CD123+ blasts. Furthermore, adult patients who achieved complete remission presented an increase in the HLA-DR MFI ratio in CD34+CD38− cells from diagnosis to second monitoring, with no significant observations in elderly patients, which would be the opposite of HLA-DR loss, which benefits the immune response [40]. The relevance of HLA-DR for LSC-like phenotypes continues to be shown, as it has been established that CD34+ cells that respond to the FLT3 ligand are mostly HLA-DR+ [41].

The ELN 2022 guidelines propose that LSC burden determination can refine risk stratification for AML patients [11]. However, few and very recent studies have implemented LSC identification in a clinical setting [21,30,32]; none have been developed with Latin American patients or have used repurposed Euroflow panels. More studies are needed across various health settings to verify the clinical efficacy of LSC identification and its association with poor prognostic factors and survival. Also, studies validating the use of LSC in day-to-day clinical settings will be necessary moving forward.

This study aims to contribute to LSC identification to aid in prognosis for health centers lacking the resources to implement the most recent guidelines. The analysis of different phenotypes within one cohort enables the selection of the phenotypes that best describe prognosis, a finding that has not previously been reported in the literature. To the best of our knowledge, this is one of the few studies to show the prognostic power of the CD123+ phenotype alone in adult and elderly individuals for AML. We also highlight how the expression of other markers can vary within potential LSC phenotypes between adult and elderly patients, which can contribute to a further step toward understanding AML in different age groups.

## 5. Conclusions

In this retrospective study, we investigated heterogeneity in acute myeloid leukemia among southern Brazilian patients using MFC analyses of distinct blast subpopulations. The main limitations of this study stem from its retrospective design, including variable and, in some analyses, limited sample sizes across subpopulations, its single-center design, and the lack of complete clinical information. In addition, the risk classification algorithm used is not the latest version, due to financial limitations in the Brazilian public health system. This study was developed to improve stratification for health centers that lack the financial support for in-depth genomic evaluation of AML patients and still rely on previous guidelines.

In conclusion, our results demonstrate that specific LSC-like phenotypes, particularly CD34+CD38−, CD123+, and CD34+CD123+ phenotypes, as well as other markers, such as CD117 and HLA-DR, are valuable prognostic indicators for AML patients, with distinct patterns across adult and elderly populations. By repositioning the widely used EuroFlow panel, our approach enhances traditional immunophenotyping by providing improved characterization of AML heterogeneity. These findings hold strong potential to refine patient risk stratification, prognostication, and personalized treatment decisions for health centers from low-income countries.

## Figures and Tables

**Table 1:** Multivariate Cox regression analysis with adult and elderly patients, normalized by age and sex (reference level = female). Different sample sizes were used for five-, three-, and one-year follow-ups.

Five-Year Follow-Up Using Diagnosis Data (n = 67)			
OS			
Factor	*p* value	HR (CI)	Other factors
CD123+% from blasts	0.02	1.024 (1.003–1.045)	no
CD34+CD38−% from total	0.002	1.084 (1.026–1.138)	no
CD34+CD38−% from blasts	0.01	1.026 (1.003–1.045)	no
RFS			
Factor	*p* value	HR (CI)	Other factors
CD123+% from blasts	0.04	1.038 (1.002–1.082)	no
CD34+CD123+% from blasts	0.04	1.092 (1.019–1.232)	no
Three-year follow-up using diagnosis data (n = 86)			
OS			
Factor	*p* value	HR (CI)	Other factors
CD117 MFI ratio from CD34+	0.02	0.987 (0.975–0.997)	Age *p* = 0.0110 HR = 1.022 (1.005–1.039)
CD34+CD38−% from total	0.006	1.064 (1.015–1.111)	Age *p* = 0.0114 HR = 1.025 (1.006–1.046)
RFS			
Factor	*p* value	HR (CI)	Other factors
CD123+% from total	0.008	1.047 (1.013–1.087)	Sex *p* = 0.0448 HR = 5.693 (1.116–35.63)
CD34 MFI ratio from CD34+CD38+	0.03	1.004 (1.000–1.009)	no
One-year follow-up using diagnosis data (n = 99)			
OS			
Factor	*p* value	HR (CI)	Other factors
CD117 MFI ratio from CD34+	0.006	0.981 (0.966–0.993)	Age *p* = 0.0002 HR = 1.038 (1.019–1.059)
CD34+CD38−% from total	0.001	1.079 (1.028–1.128)	Age *p* = 0.0006 HR = 1.038 (1.017–1.062)
RFS			
Factor	*p* value	HR (CI)	Other factors
HLA-DR MFI ratio from CD34+CD123+	0.01	0.092 (0.01–0.446)	no
CD34 MFI ratio from CD34+CD38+	0.02	1.005 (1.001–1.010)	no
CD34 MFI ratio from CD34−CD38+	0.02	1.705 (1.022–2.905)	no
NG2 MFI ratio from CD34+CD38+	0.02	1.138 (1.013–1.294)	no

## Data Availability

The original contributions presented in this study are included in the article. Further inquiries can be directed to the corresponding author.

## Supplementary Materials

The following supporting information can be downloaded at: https://www.mdpi.com/article/10.3390/cancers18091394/s1, Table S1: Sample size and characteristics for each of the analyses performed (number of patients); Table S2: Tubes used for this study from the EuroFlow AML/MDS antibody panel. Adapted from Kalina et al. [14]; Table S3: Parameters evaluated in the analysis of each phenotype; Figure S1: Gating strategy applied to identify and isolate each blast/immature cell subpopulation. Erythroblasts are in bright green, neutrophils and eosinophils are in deep blue, monocytes are in light blue, lymphocytes are in deep green, and blasts/immature cells are in magenta (when using tube 5, it is possible to separate plasma cells by CD38 expression and when using tube 6, it is possible to separate mastocytes and basophils through CD203 and CD123 expressions); Table S4: Correlation analysis in adults and elderly patients using DX data (Spearman r test was applied); Figure S2: General overall survival (OS) and relapse-free survival (RFS) information for adults, elders, and transplant patients. (a) Five-year overall survival for adults (b) Three-year overall survival for adults (c) Five-year relapse-free survival for adults (d) Three-year relapse-free survival for adults (e) Five-year overall survival for elders (f) Three-year overall survival for elders (i) Three-year overall survival comparing transplant and non-transplant patients (j) Three-year relapse-free survival comparing transplant and non-transplant patients (g) Percentage of blasts as first monitoring (day 30) comparison between transplant and alive non-transplant patients (h) Percentage of blasts at end-of-induction monitoring (day 90) comparison between transplant and alive non-transplant patients. It was not possible to calculate the median time-to-event for RFS in elderly patients due to the small sample size of relapsing patients; Figure S3: CD36+ and CD123+ subpopulations associations with prognostic parameters at diagnosis. (a) CD34 MFI ratio from CD36+ blasts (Kruskal–Wallis test between adult risk levels *p* = 0.005, Dunn’s post hoc test between adults at intermediate and high risk levels *p* = 0.02); (b) CD117 MFI ratio from CD36+ blasts (Kruskal–Wallis test between adult risk levels *p* = 0.01, Dunn’s post hoc test between adults at standard and intermediate risk levels *p* = 0.05); (c) CD36 MFI ratio from CD36+ blasts (Mann–Whitney test between relapse and non-relapse adults *p* = 0.02); (d) CD34 MFI ratio from CD36+ blasts (Mann–Whitney test between relapse and non-relapse adults *p* = 0.04, Mann-Whitney test between non-relapse adults and elders *p* = 0.03); (e) CD34 MFI ratio from CD123+ blasts (Kruskal–Wallis test between adult risks *p* = 0.04, Dunn’s post hoc test between adults from standard and high-risk *p* = 0.06); (f) ROC curve for the CD34 MFI ratio from CD123+ blasts comparing standard– and high-risk adults (*p* = 0.02).

## Abbreviations

The following abbreviations are used in this manuscript:

AML	Acute myeloid leukemia
CI	Confidence interval
CR	Complete remission
ELN	European Leukemia Net
FLT3-ITD	FMS-like tyrosine kinase 3 internal tandem duplications
FSC	Forward scatter
HCPA	Hospital de Clínicas de Porto Alegre
HR	Hazard ratio
HSC	Hematopoietic stem cells
LGPD	Lei geral de proteção de dados (general law of data protection, in Portuguese)
LSC	Leukemia stem cells
MFC	Multiparametric flow-cytometry
MFI	Mean fluorescence intensity
MRD	Measurable residual disease
n-CR	Non-complete remission
OS	Overall survival
RFS	Relapse-free survival
SSC	Side scatter
WHO	World Health Organization
