# Supplementary material for: LSC-like Phenotypes Aid in the Prognosis of Adult and Elderly Acute Myeloid Leukemia Patients at a Resource-Limited Health Center
Source: Cancers (Basel). 2026 Apr 28;18(9):1394. doi: 10.3390/cancers18091394 (PMC13162642; doi:10.3390/cancers18091394)
Supplement: Supplementary file 1 [file cancers-18-01394-s001.zip › cancers-4233398-supplementary.pdf]

Table S1. Sample size and characteristics for each of the analyses performed (number of patients).

Characteristic		All patients	CD34+	CD36+	CD34+ CD36+	CD123+	CD34+ CD123+	CD34&CD38
Risk								
	Standard	12	12	9	9	3	3	7
	Intermediate	26	26	15	15	12	9	19
	High	61	61	23	23	22	16	46
Median age in year (range)		Number of patients						
45 (21-59)	Adults <60	57	57	29	29	26	21	45
68 (60-89)	Elderly ≥60	42	42	18	18	11	7	27
Biological sex								
	Female	41	41	20	20	15	12	29
	Male	58	58	27	27	22	16	43
FLT3/ITD alteration								
	Positive	19	19	8	8	9	7	17
	Negative	47	47	27	27	22	18	33
	Missing	33	33	12	12	6	3	22
Treatment response								
	Complete remission	37	37	19	19	16	13	29
	Non-complete remission	24	24	13	13	11	9	18
	NA	38	38	15	15	10	6	25
Relapse								
	Yes	28	28	14	14	16	14	22
	No	25	25	16	16	7	6	19

	NA	46	46	17	17	14	8	31
Allo-HCT Transplant								
	Yes	9	9	5	5	5	5	7
	No	90	90	42	42	32	23	65
AML subtype								
	immature	23	23	10	10	10	7	16
	myelomonocytic	17	17	9	9	6	5	13
	monocytic	28	28	13	13	10	5	22
	unspecified	31	31	15	15	11	11	21

NA: not available.

Table S2. Tubes used for this study from the EuroFlow AML/MDS antibody panel. Adapted from Kalina et al. [14].

Diagnosis

Tube	PacB	PacO	FITC	PE	PerCPCy5.5	PECy7	APC	APCH7
3	HLA-DR	CD45	CD36	CD105	CD34	CD117	CD33	CD71
5	HLA-DR	CD45	CD15	NG2	CD34	CD117	CD22	CD38
6	HLA-DR	CD45	CD42a and CD61	CD203c	CD34	CD117	CD123	CD4

Minimal Residual Disease

Tube	PacB	PacO	FITC	PE	PerCPCy5.5	PECy7	APC	APCH7
1	HLA-DR	CD45	cyMPO	CD64	CD34	CD117	CD33	CD38

HLA-DR (RRID:AB_2632616), CD45 (RRID:AB_2870319), CD36 (RRID:AB_2870400), CD15 (RRID:AB_2868627), CD42a (RRID:AB_400374), CD61 (RRID:AB_2868851), cyMPO (RRID:AB_2917957), CD105 (RRID:AB_2033932), NG2 (AB_3678969), CD203c (RRID:AB_141295), CD64 (RRID:AB_1727085), CD34 (RRID:AB_2868843), CD117 (RRID:AB_131184), CD33 (RRID:AB_2868824), CD22 (RRID:AB_2868646), CD123 (RRID:AB_2870447), CD71 (RRID:AB_2870381), CD38 (RRID:AB_2870413), CD4 (RRID:AB_1645732).

Table S3. Parameters evaluated in the analysis of each phenotype.

Parameter/ Analysis	CD34+	CD36+	CD34+CD36+	CD123+	CD34+CD123+	CD34&CD38
Percentages analyzed	CD34+ blasts from total cells, CD34+ from total blasts	CD36+ blasts from total cells, CD36+ from total blasts	CD34+CD36+ blasts from total cells, CD34+CD36+ from total blasts	CD123+ blasts from total cells, CD123+ from total blasts	CD34+blasts from total cells, CD34+CD123+ blasts from total cells, CD34+CD123+ from total blasts	CD34+CD38-, CD34+CD38+, CD34-CD38+, CD34-CD38- blasts from total cells and from total blasts
How MFI ratio was calculated	CD34+ blasts MFI/ CD34- blasts MFI	CD36+ blasts MFI/ CD36- blasts MFI	CD34+CD36+ blasts MFI/ CD34+CD36-, CD34-CD36+, CD34-CD36- combined blasts MFI	CD123+ blasts MFI/ CD123- blasts MFI	CD34+123+ blasts MFI/ CD34+CD123-, CD34-CD123+, CD34-CD123- combined blasts MFI	CD34+CD38-, or CD34+CD38+, or CD34-CD38+ blasts MFI/ CD34-CD38- blasts MFI
MFI ratios analyzed	CD33, CD34, CD36, CD117, HLA-DR	CD33, CD34, CD36, CD117, HLA-DR	CD33, CD34, CD36, CD117, HLA-DR	CD34, CD117, CD123, HLA-DR	CD34, CD117, CD123, HLA-DR	CD34, CD38, CD117, HLA-DR, NG2

Only CD34+ and CD34&CD38 subpopulations were analyzed at diagnosis, day 30, and day 90 of monitoring. All others were analyzed only at diagnosis.

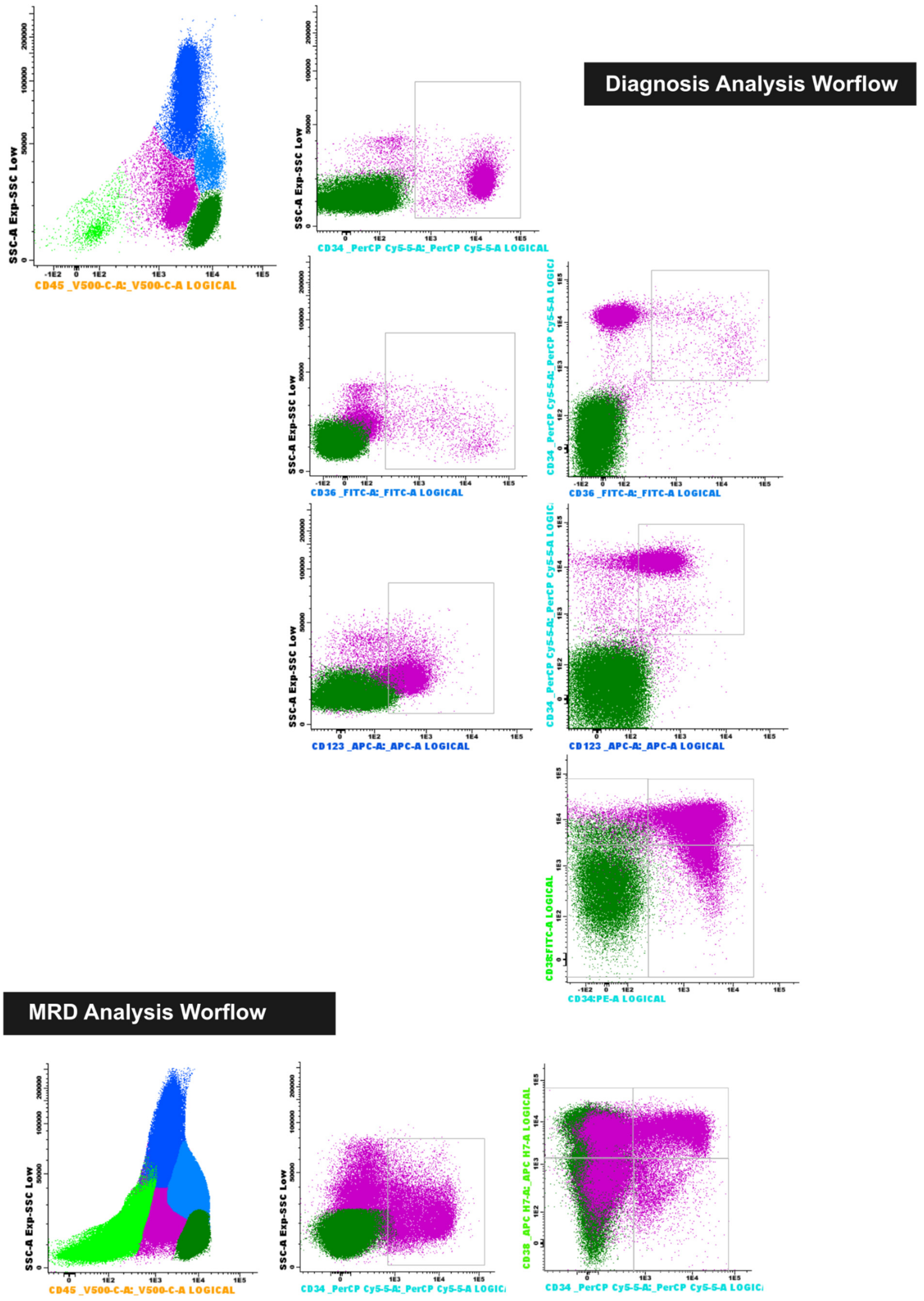

Figure S1. Gating strategy applied to identify and isolate each blast/immature cell subpopulation. Erythroblasts are in bright green, neutrophils and eosinophils are in deep blue, monocytes are in light blue, lymphocytes are in deep green, and blasts/immature cells are in magenta (when using tube 5, it is possible to separate plasma cells by CD38 expression and when using tube 6, it is possible to separate mastocytes and basophils through CD203 and CD123 expressions).

Table S4. Correlation analysis in adults and elderly patients using DX data (Spearman r test was applied).

5-year follow-up (n = 66)		
**Correlation**	p-value	Spearman R
Age and OS	0,008	-0,323
Age and CD123+% from total	0,001	-0,692
Age and CD123+% from blasts	0,026	-0,524
Age and CD123 MFI ratio from CD123+	0,001	0,738
Age and CD34+CD123+% from total	0,005	-0,800
Age and CD34+CD123+% from blasts	0,048	-0,618
Age and CD123 MFI ratio from CD34+CD123+	0,014	0,727
Age and CD117 MFI ratio from CD34+CD123+	0,020	-0,700
RFS and NG2 MFI ratio from CD34+CD38-	0,029	0,852
OS and CD123 MFI ratio from CD34+CD123+	0,028	0,671
**3-year follow-up (n = 86)**		
**Correlation**	p-value	Spearman R
Age and OS	0,004	-0,310
Age and RFS	0,006	-0,298
Age and CD123 MFI ratio from CD123+	0,002	0,541
Age and CD34+CD123+% from total	0,015	-0,524
Age and CD34 MFI ratio from CD34+CD123+	0,022	-0,510
Age and CD123 MFI ratio CD34+CD123+	0,004	0,612
Age and CD34+CD38+% from total	0,040	-0,268
Age and HLA-DR MFI ratio from CD34+CD38+	0,004	-0,508
RFS and CD117 MFI ratio from CD34+	0,018	0,267
RFS and HLADR MFI ratio from CD34+	0,049	0,218
RFS and CD34+CD123+% from blasts	0,050	-0,444
RFS and CD123 MFI ratio from CD34+CD123+	0,016	0,546
RFS and HLADR MFI ratio from CD34+CD123+	0,010	0,573
RFS and CD34-CD38+% from blasts	0,046	-0,263
RFS and HLADR MFI ratio from CD34+CD38+	0,039	0,386
RFS and HLADR MFI ratio from CD34-CD38+	0,042	0,380
RFS and CD117 MFI ratio from CD34+CD36+	0,037	0,358
OS and CD117 MFI ratio from CD34+	0,008	0,293
OS and CD34+CD123+% from blasts	0,049	-0,435
OS and CD123 MFI ratio from CD34+CD123+	0,035	0,473
OS and HLADR MFI ratio from CD34+CD123+	0,013	0,546
OS and HLADR MFI ratio from CD34-CD38+	0,035	0,387
**1-year follow-up (n = 99)**		
**Correlation**	p-value	Spearman R
Age and OS	0,00007	-0,388
Age and RFS	0,001	-0,325
Age and CD123 MFI ratio from CD123+	0,011	0,416
Age and CD34+CD123+% from total	0,002	-0,551
Age and CD34 MFI ratio from CD34+CD123+	0,017	-0,462
Age and HLA-DR MFI ratio from CD34+CD38+	0,015	-0,367
Age and HLA-DR MFI ratio from CD34-CD38+	0,031	-0,328
RFS and CD117 MFI ratio from CD34+	0,014	0,254
RFS and CD123 MFI ratio from CD34+CD123+	0,022	0,447
RFS and HLADR MFI ratio from CD34+CD123+	0,011	0,493
RFS and CD117 MFI ratio from CD34+CD36+	0,039	0,306
OS and CD117 MFI ratio from CD34+	0,013	0,258
OS and CD33 MFI ratio from CD34+	0,031	-0,302
OS and CD123 MFI ratio from CD34+CD123+	0,018	0,459

OS and HLADR MFI ratio from CD34+CD123+	0,027	0,432
OS and CD34+CD38-% from blasts	0,031	-0,254
OS and CD34 MFI ratio from CD34+CD38+	0,048	0,238

RFS: relapse-free survival, OS: overall survival.

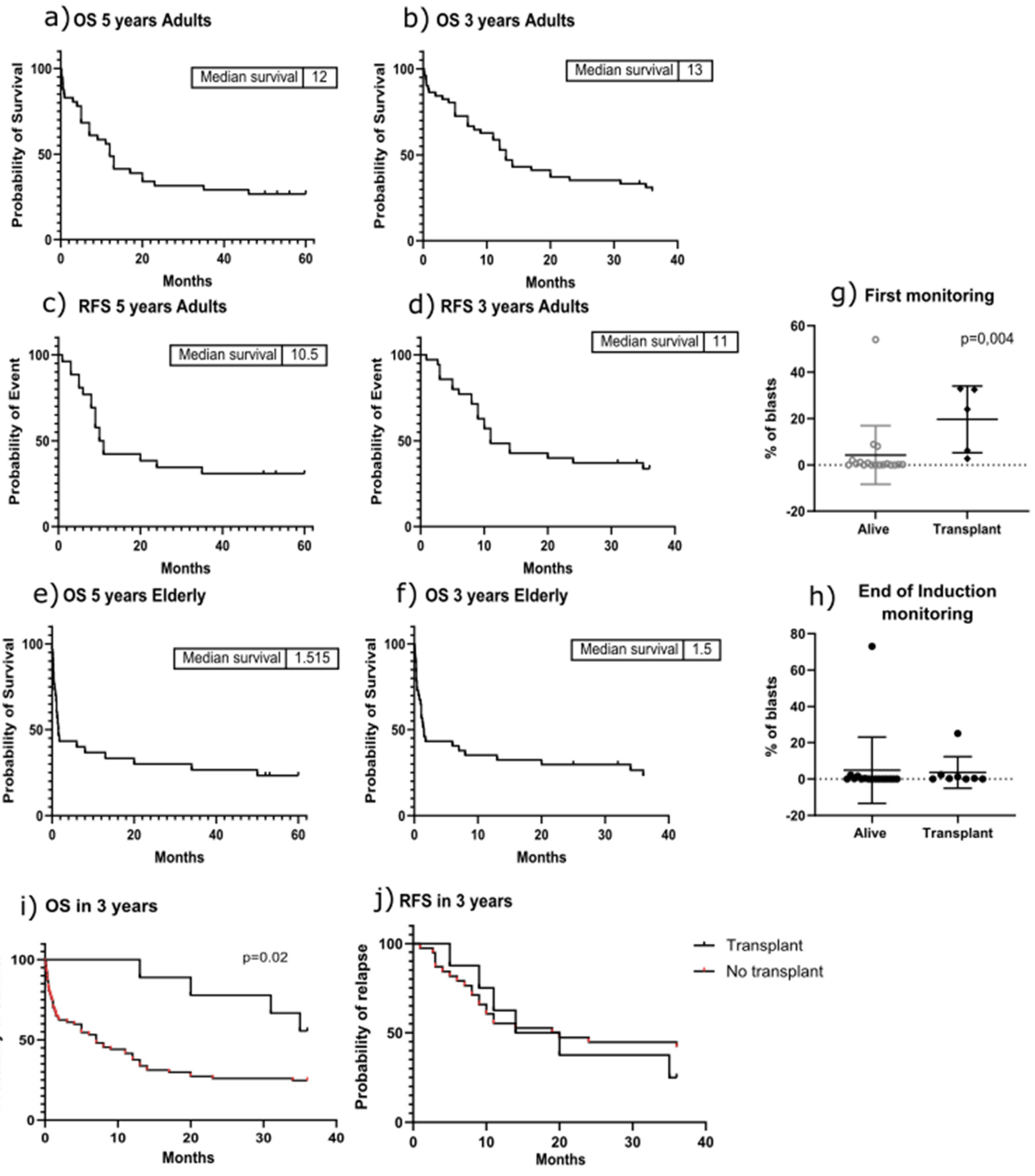

Figure S2. General overall survival (OS) and relapse-free survival (RFS) information for adults, elders, and transplant patients. (a) Five-year overall survival for adults (b) Three-year overall survival for adults (c) Five-year relapse-free survival for adults (d) Three-year relapse-free survival for adults (e) Five-year overall survival for elders (f) Three-year overall survival for elders (i) Three-year overall survival comparing transplant and non-transplant patients (j) Three-year relapse-free survival comparing transplant and non-transplant patients (g) Percentage of blasts as first monitoring (day 30) comparison between transplant and alive non-transplant patients (h) Percentage of blasts at end-of-induction monitoring (day 90) comparison between transplant and alive non-transplant patients. It was not possible to calculate the median time-to-event for RFS in elderly patients due to the small sample size of relapsing patients.

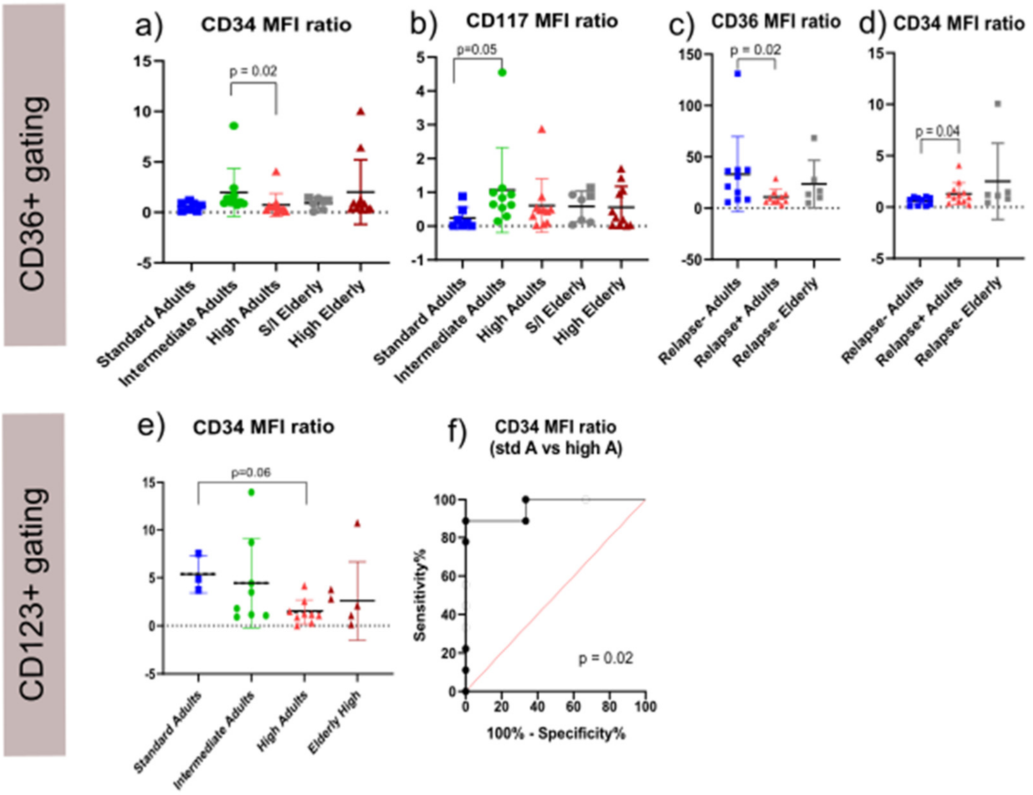

Figure S3. CD36+ and CD123+ subpopulations associations with prognostic parameters at diagnosis. (a) CD34 MFI ratio from CD36+ blasts (Kruskal–Wallis test between adult risk levels p = 0.005, Dunn's post hoc test between adults at intermediate and high risk levels p = 0.02); (b) CD117 MFI ratio from CD36+ blasts (Kruskal–Wallis test between adult risk levels p = 0.01, Dunn's post hoc test between adults at standard and intermediate risk levels p = 0.05); (c) CD36 MFI ratio from CD36+ blasts (Mann–Whitney test between relapse and non-relapse adults p = 0.02); (d) CD34 MFI ratio from CD36+ blasts (Mann–Whitney test between relapse and non-relapse adults p = 0.04, Mann-Whitney test between non-relapse adults and elders p = 0.03); (e) CD34 MFI ratio from CD123+ blasts (Kruskal–Wallis test between adult risks p = 0.04, Dunn's post hoc test between adults from standard and high-risk p = 0.06); (f) ROC curve for the CD34 MFI ratio from CD123+ blasts comparing standard– and high-risk adults (p = 0.02).
